# Subclinical sympathetic neuropathy appears early in the course of Crohn's disease

**DOI:** 10.1186/1471-230X-7-33

**Published:** 2007-08-14

**Authors:** Bodil Ohlsson, Göran Sundkvist, Stefan Lindgren

**Affiliations:** 1 Department of Clinical Sciences, Gastroenterology Division, Entrance 35, 205 02 Malmö, Lund University, Sweden; 2 Department of Clinical Sciences, Diabetes Epidemiology and Neuropathy Division, Entrance 51, 205 02 Malmö, Lund University, Sweden; 3 deceased

## Abstract

**Background:**

We have previously demonstrated that patients with Crohn's disease (CD) of long duration have signs of autonomic neuropathy. The aim of this study was to examine whether autonomic neuropathy is an early manifestation of CD, or a sign appearing late in the course.

**Methods:**

Twenty patients, median age 40 years, with a short duration of CD were included. Examination of autonomic reflexes included heart rate reaction to tilt (acceleration index – AI, brake index – BI) and heart rate variation to deep-breathing (expiration/inspiration index-E/I). Seven years later the same examinations were repeated, and in addition we examined the vasoconstriction response to indirect cooling by laser Doppler (vasoconstriction-index – VAC-index). The results were compared with healthy individuals.

**Results:**

There was no difference in the blood pressure between controls and the patients with CD at rest, but eight minutes after tilt, the systolic blood pressure was lowered in patients compared to controls, both at the first assessment (p = 0.016) and after seven years (p = 0.042). The change in systolic blood pressure between rest and eight minutes after tilt was not significant at the first assessment, while a significant change compared to controls was observed seven years later (p = 0.028). This indicates a progressive dysfunction. There were no differences in E/I, AI, BI or VAC indexes between patients and controls.

**Conclusion:**

Patients with CD suffer from autonomic neuropathy early in their disease, suggesting involvement of many different organ systems in this entity.

## Background

Crohn's disease (CD) is a chronic inflammatory condition that may involve the entire gastrointestinal tract. Both infectious agents and autoimmune mechanisms have been proposed as aetiological factors. The inflammatory process is transmural and extra intestinal inflammatory manifestations are common, suggesting a systemic character of this disorder [1].

We have earlier described the presence of autonomic neuropathy in both CD and ulcerative colitis (UC), although more prevalent in CD [2,3]. Parasympathetic dysfunction in terms of low expiratory/inspiratory index (E/I Index) and acceleration index (AI) was most prevalent in patients with UC, while sympathetic dysfunction in terms of low brake index (BI) dominated in patients with CD. It has never been described whether it is the engagement of the enteric nervous system (ENS) located in the intestinal wall [4], which is a part of the autonomic nervous system, which leads to autonomic dysfunction or if other parts of the autonomic nervous system may be primarily affected.

The former studies examined patients who had suffered from their disease for many years [2,3]. We now wanted to compare autonomic nerve functions in controls and patients with CD of a shorter duration at two different occasions, to find out if neuropathy is a concomitant manifestation of the disease, or a complication that develops many years later.

## Methods

### Patients

Twenty patients (14 women) with CD of the small intestine and/or colon were invited to participate in this study examining autonomic nerve functions. The median age was 40 years (interquartile ranges [IQR], 25–63 years), and the median duration of disease 44 months (IQR, 28–50 months). The diagnosis was based on established clinical, radiological and/or endoscopic and histological criteria [5]. Seven years later, 15 of the patients were re-examined with the same autonomic tests and blood samples (see below). The median age was then 36 years (IQR, 31–59 years). Three of the patients had died during these years (from stroke, pneumonia and peritonitis, respectively), and one was too sick in Parkinson's disease to perform the experiments. One had moved from the region. All patient records were scrutinized to evaluate incidents in their medical history during the last years, and the patients were also interviewed. The clinical data were obtained at the time of the examinations of autonomic neuropathy (Table 1).

**Table 1:** Characteristics of patients with Crohn's disease at the time of examinations

	**First assessment**		**Second assessment**	
	Number (20)	%	Number (15)	%

**Disease distribution**				
Distal ileum	2	10	1	7
Colon	5	25	5	33
Colon and small intestine	6	30		
**Activity of disease**				
Inactive	18	90	13	87
Low activity	2	10	2	13
**Operations**				
Not operated	8	40	4	27
Small intestinal resection	1	5		
Ileocekal resection	8	40	8	53
Partial colectomy	2	10	1	7
Complete colectomy			1	7
Small intestinal resection and partial colectomy			1	7
**Medication**				
Sulfasalazine, 5-ASA	7	35	6	40
Immunosuppressive drugs (azathioprine, 6-MP, methotrexate, TNFα)	5	25	6	40
Steroids	3	15	2	13

Besides the intestinal disease, some patients suffered from extra intestinal manifestations or had functional symptoms associated with inflammatory bowel disease. None of the patients suffered from classical symptoms of autonomic neuropathy (Table 2). Of the 15 patients who remained after seven years, no one suffered from diabetes mellitus, but one patient had autoimmune thyreoditis, one Mb Bechterew, one a prolactinom and one urolithiasis in their medical history. One of the patients had suffered from serious depression and had used antidepressant drugs for several years, whereas the other patients did not suffer from psychosocial problems or used any drugs that could affect the nervous system.

**Table 2:** Extra intestinal and functional bowel symptoms in patients with Crohn's disease

	**First assessment**		**Second assessment**	
**Symptoms**	Number (20)	%	Number (15)	%

Skin diseases	3	15	6	40
Arthritis, arthralgia	3	15	7	50
Ocular diseases	1	5	4	30
Irritable Bowel Disease (IBS)	8	40	10	70
Dyspepsia	6	30	4	30
Autonomic neuropathy	-	-	-	-

### Autonomic nerve function tests

#### Orthostatic tilt table test

The subject was tilted rapidly (two seconds) to the upright position (90°), and the immediate heart rate change (normally an immediate acceleration followed by a transient deceleration) were recorded on the electrocardiogram (ECG), and the AI and BI were calculated, on the basis of the R-R intervals [6]. The blood pressure was measured before the tilt, and then every minute up to eight minutes after the tilt. The AI and BI evaluate not only the parasympathetic nervous tone but also the sympathetic nerve function [6,7].

#### Deep breathing test

The subject performed six maximal expirations and inspirations in the supine position during recording of a continuous ECG. The E/I ratio was calculated from the mean value of the longest R-R interval during expiration and the shortest R-R interval during inspiration. This is an established test of vagal, parasympathetic nerve function [8].

#### Laser Doppler Perfusion Imaging (LDPI)

Skin perfusion was obtained with a PIM 1.0 instrument (Perimed AB, Stockholm, Sweden), which measures the Doppler shift of backscattered light from the skin surface, using a He-Ne laser light source. The mean blood flow during two minutes preceding indirect cooling (LDPI_h_) was used as a reference against which the lowest value of perfusion during the first minute of cooling (LDPI_c_) was compared. A numerical expression of the response of blood flow to indirect cooling was obtained by constructing an index for vasoconstriction (VAC = LDPI_c_/LDPI_h_), which was expressed in age-corrected Z-scores [9]. This is a measure of sympathetic nerve function [10]. This test was only performed at the second assessment.

### Interpretation of autonomic test results

A previously carefully characterized reference group comprising 56 healthy subjects (22 female), aged 16–59 years (median 39 years) [11] acted as controls regarding E/I ratio, AI and BI results, whereas a control group comprising 80 healthy subjects (37 female), aged 19–81 years (median 59 years) [9] was used for comparison regarding the VAC-index.

To match for age, test results were expressed in age-related values – that is standard deviation (SD), as previously described [12]. An age-related value below -1.64 SD (one-sided test, 95% confidence interval) was considered as abnormal for the tests determining E/I ratio, AI and BI [13). Concerning the VAC-index, high Z-scores, >1.64 SD above controls are abnormal [9].

### Biochemical tests of inflammation and malabsorption

These included a full blood count, vitamins (cobalamines, folic acid) and trace elements (chloride, potassium, calcium and magnesium), blood glucose, albumin, sedimentation rate, C-reactive protein (CRP), thyroid stimulating hormone and serum enzymes. All blood tests were performed at the Department of Clinical Chemistry, in accordance with routine procedures. The tests were used to assess the disease activity and to exclude any other aetiology to abnormal nerve function tests.

### Statistical analyses

Values are given as median [IQR]. Mann-Whitney U test was used for comparisons between the controls and the patients at two different occasions. Fishers' exact test was used to compare the frequencies of findings. As 25% of the patients were lost for follow up, no longitudinal calculations were performed. P < 0.05 was considered to indicate statistical significance.

### Ethical consideration

This prospective study was approved by the Ethics Committee at Lund University. All patients gave written informed consent before entering the study.

## Results

### Orthostatic tilt table test

There was no difference in systolic blood pressure between controls and patients during rest. In the controls, the systolic blood pressure was equal at rest and one and eight minutes (125 [115–135], 125 [120–135] and 125 [115–136] mg Hg, respectively) after tilt. In contrast, patients with CD had lower blood pressure eight minutes after tilt at the first assessment compared to controls (112 [105–125] mm Hg, p = 0.016). At the second assessment after seven years, there was a tendency to lower blood pressure one minute after tilt compared to controls (118 [105–135] mm Hg, p = 0.054), and after eight minutes, the blood pressure was further lowered (115 [110–125] mm Hg, p = 0.042).

**Figure 1:**
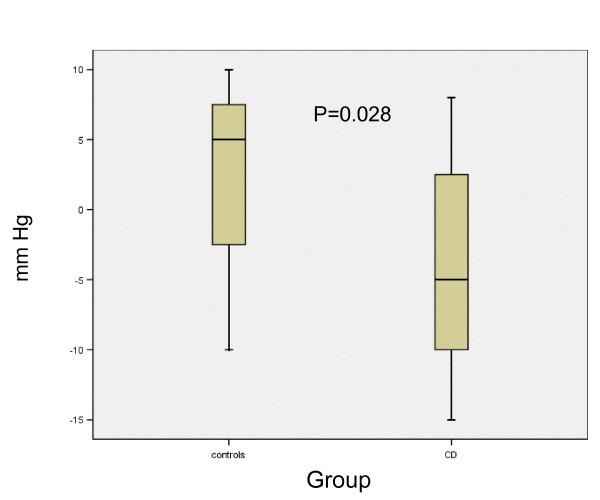
The change in systolic blood pressure eight minutes after tilt compared to rest at the second assessment in healthy controls and in patients with Crohn's disease (CD).

When calculating the change in systolic blood pressure between rest and after one and eight minutes, this did not differ at the first assessment compared to controls (data not shown). However, at the second assessment the systolic blood pressure tended to differ after one minute (p = 0.063), and the change was significantly different compared to controls after eight minutes (Fig 1). Thus, the changes suggesting sympathetic neuropathy were more pronounced after seven years. There was no difference between controls and patients in AI or BI at any assessment (data not shown). There was no association between frequent relapses, severe disease or use of aggressive immune modulating drugs and development of neuropathy (data not shown).

### Deep breathing test and Laser Doppler Perfusion Imaging (LDPI)

There were no differences in E/I or VAC indexes between controls and patients with CD, neither at the first nor at the second assessment (data not shown).

## Discussion

The present study disclosed an impaired ability to keep the systolic blood pressure after tilt in patients with a short duration of CD. This is in accordance with early sympathetic neuropathy [14]. Autonomic neuropathy already early in the course of CD supports the concept of CD as a systemic disease not only affecting the gastrointestinal tract but also other organ systems. As the changes were more pronounced after seven years than at the first assessment, and as abnormal indexes are evident after even longer disease duration [2], there seems to be a progression of the autonomic neuropathy, independently of the disease activity.

In CD, proliferate and injurious changes have been described in the enteric smooth muscles, enteric nerves and interstitial cells of Cajal (ICCs) [15-19]. We have recently found that half of the patients who suffer from CD also have ganglioneuritis and abnormal ICCs examined by conventional histopathological evaluation and immunostaining for ICCs and T-cells [4]. Ganglioneuritis correlated with higher values of E/I and BI [4]. We do not know what this means, and if elevated values also may indicate neuropathy. Among the patients in the current study, eight were operated and the resected specimens were evaluated for damage to the enteric nervous system (ENS). Although one or two patients had E/I, AI or BI below reference value at each assessment, no one who had enteric neuropathy in routine histopathology combined with immunohistochemistry showed pathological low values in the autonomic nerve function tests. Thus, the absence of pathological results in these tests may coexist with histological ganglioneuritis, and does not exclude autonomic neuropathy. Evaluation of systolic blood pressure may thus be a more sensitive examination than the other tests in this patient population. Besides possible differences in the prevalence of ganglioneuritis, which has not been examined earlier, the longer disease duration in our previous study [2] may explain the different results in indexes between our two studies.

As the ENS is part of the autonomic nervous system, damage to ENS could reflect a general damage to the nervous system. The current findings are in accordance with a recent description of sympathetic neuropathy in patients with chronic intestinal pseudo-obstruction (CIPO) [20], a disease characterised by pronounced neuropathic changes in the ENS. Furthermore, CIPO has been shown to coexist with CD [21]. Altogether, some cases of idiopathic CIPO and CD may represent the same disease entity with damage both to the ENS and the sympathetic nervous system [2,4,20,21].

Although the heart frequency and the diastolic blood pressure increased, the systolic blood pressure was lowered after tilt. VAC index, which also reflects the sympathetic nervous system [10], was within normal range in this study. However, this test reflects the reaction to cooling, whereas the orthostatic tilt test reflects the baro reflex function [22]. Thus, our findings suggest disturbed baro receptor reflexes rather than failure of vasoconstriction as an early feature of sympathetic neuropathy in CD.

The bi-directional brain-gut pathways transmit environmental signals to the intestine, and in the same way signals from the intestine to the brain. Many different signals from the central nervous system, e.g. stress, could thus modulate inflammatory bowel diseases [23]. In addition, the hypothalamic-pituitary-adrenal axis and the sympathetic-adrenal medullar axis also modulate gut secretory, absorptive and defensive factors. Only a few studies are performed to examine the function of the autonomic nervous system in patients with inflammatory bowel diseases [2,3]. In experimental animal studies, the autonomic nervous system has immune modulating effects on the gastrointestinal mucosa and affects the development of colitis [24-26]. Sympathetic innervation of the colon was lost in colitis animals, which modulated the cytokine secretion [24]. Thus, during both healthy and inflamed states, there seems to be a strong relation between the gastrointestinal tract and the central and autonomic nervous system. These associations need to be further studied to understand the mechanisms involved in the development of colitis and autonomic neuropathy.

It should be noted that nobody of our patients with CD had classical symptoms of autonomic neuropathy [27], although autonomic nerve function tests were abnormal in some patients. However, a lack of symptoms in spite of autonomic neuropathy is frequent in diabetes mellitus [28]. The neuropathy per se may contribute to defect signalling to the central nervous system, and subsequently absence of symptoms. However, after several years the sympathetic neuropathy may have clinical impacts on the symptomatology and care. Autonomic neuropathy may lead to postural hypotension, impotence, swallowing disturbances and gastrointestinal dysmotility [23,29]. The disturbances in the ENS and the other parts of the autonomic nervous system in inflammatory bowel diseases may lead to altered bowel function very difficult to differ from an inflammatory relapse [30], and obscure the choice of treatment.

## Conclusion

Our data suggest that autonomic sympathetic neuropathy is an early systemic feature of CD independent of disease activity. The neuropathy was slightly more pronounced after seven years and this progressive course is consistent with the more pronounced autonomic neuropathy manifested by abnormal indexes previously demonstrated in patients with CD of longer duration [2].

## Competing interests

The authors declare that they have no competing interests.

## Authors' contributions

BO participated in the design of the study, recruited subjects, financed the study and drafted the manuscript. GS and SL participated in the design of the study, and participated in drafting of the manuscript.

## Pre-publication history

The pre-publication history for this paper can be accessed here:



## References

[B1] Podolsky DK (2002). Inflammatory bowel disease. N Engl J Med.

[B2] Lindgren S, Lilja B, Rosén I, Sundkvist G (1991). Disturbed autonomic nerve function in patients with Crohn's disease. Scand J Gastroenterol.

[B3] Lindgren S, Stewenius J, Sjölund K, Lilja B, Sundkvist G (1993). Autonomic vagal nerve dysfunction in patients with ulcerative colitis. Scand J Gastroenterol.

[B4] Ohlsson B, Veress B, Lindgren S, Sundkvist G (2007). Enteric ganglionitis and ganglioneuritis and abnormal interstitial cells of Cajal; features of inflammatory bowel disease. Inflammatory Bowel Diseases.

[B5] Lennard-Jones JE (1989). Classification of inflammatory bowel disease. Scand J Gastroenterol Suppl.

[B6] Sundkvist G, Lilja B, Almer LO (1980). Abnormal diastolic blood pressure and heart rate reactions to tilting in diabetes mellitus. Diabetologia.

[B7] Bergström B, Manhem P, Bramnert M, Lilja B, Sundkvist G (1989). Impaired responses of plasma catecholamines to exercise in diabetic patients with abnormal heart rate reactions to tilt. Clin Physiol.

[B8] Sundkvist G, Almer LO, Lilja B (1979). Respiratory influence on heart rate in diabetes mellitus. Br Med J.

[B9] Bornmyr S, Svensson H, Söderström T, Sundkvist G, Wollmer P (1998). Finger skin blood flow in response to indirect cooling in normal subjects and in patients before and after sympathectomy. Clin Physiol.

[B10] Freccero C, Svensson H, Bornmyr S, Wollmer P, Sundkvist G (2004). Sympathetic and parasympathetic neuropathy are frequent in both type 1 and type 2 diabetic patients. Diabetes Care.

[B11] Bergström B, Lilja B, Rosberg K, Sundkvist G (1986). Autonomic nerve function tests. Reference values in healthy subjects. Clin Physiol.

[B12] Bergström B, Lilja B, Österlin S, Sundkvist G (1990). Autonomic neuropathy in non-insulin dependent (typ II) diabetes mellitus. Possible influence of obesity. J Intern Med.

[B13] Forsen A, Kangro M, Sterner G, Norrgren K, Thorsson O, Wollmer P, Sundkvist G (2004). A 14-year prospective study of autonomic nerve function in type 1 diabetic patients: association with neuropathy. Diabet Med.

[B14] Gibbons CH, Freeman R (2006). Delayed orthostatic hypotension. A frequent cause of orthostatic intolerance. Neurology.

[B15] Bishop AE, Polak JM, Bryant MG, Bloom SR, Hamilton S (1980). Abnormalities of vasoactive intestinal polypeptide containing nerves in Crohn's disease. Gastroenterology.

[B16] Dvorak AM, Osage JE, Monahan RA, Dickersin GR (1980). Crohn's disease: Transmission electron microscopic studies. III. Target tissues. Proliferation of and injury to smooth muscle and the autonomic nervous. Hum Pathology.

[B17] Sjölund K, Schaffalitzky de Muckadell OB, Fahrenkrug J, Håkansson R, Peterson BG, Sundler F (1983). Peptide containing nerve fibres in the gut wall in Crohn's disease. Gut.

[B18] Geboes K, Collins S (1998). Structural abnormalities of the nervous system in Crohn's disease and ulcerative colitis. Neurogastroenterol Mot.

[B19] Porcher C, Baldo M, Henry M, Orsoni P, Julé Y, Ward SM (2002). Deficiency of interstitial cells of Cajal in the small intestine of patients with Crohn's disease. Am J Gastroenterol.

[B20] Mattsson T, Roos R, Sundkvist G, Valind S, Ohlsson B (2007). Sympathetic nerve dysfunction is common in patients with enteric dysmotility. J Clin Gastroenterol.

[B21] Ohlsson B, Veress B, Fork F-T, Toth E (2005). Coexistent chronic idiopathic intestinal pseu doobstruction and inflammatory bowel disease. Gut.

[B22] Vogel ER, Sandroni P, Low PA (2005). Blood pressure recovery from valsalva maneuver in patients with autonomic failure. Neurology.

[B23] Collins SM (2001). Stress and the gastrointestinal tract IV. Modulation of intestinal inflammation by stress: basic mechanisms and clinical relevance. Am J Physiol Gastrointest Liver Physiol.

[B24] Straub RH, Stebner K, Härle P, Kees F, Falk W, Schölmerich J (2005). Key role of the sympathetic microenvironment for the interplay of tumour necrosis factor and interleukin 6 in normal but not in inflamed mouse colon mucosa. Gut.

[B25] Ghia JE, Blennerhassett P, Kumar-Ondiveeran H, Verdu EF, Collins SM (2006). The vagus nerve: A tonic inhibitory influence associated with inflammatory bowel disease in a murine model. Gastroenterology.

[B26] Saunders PR, Miceli P, Vallance BA, Wang L, Pinto S, Tougas G, Kamath M, Jacobson K (2006). Noradrenergic and cholinergic neural pathways mediate stress-induced reactivation of colitis in the rat. Autonomic Neuroscience: Basic & Clinical.

[B27] Bannister R, Bannister R (1983). Clinical features of progressive autonomic failure. Autonomic failure A textbook of clinical disorders of the autonomic nervous system.

[B28] Kennedy WR, Navarro X, Sakuta M, Mandell H, Knox CK, Sutherland DER (1989). Physiological and clinical correlates of cardiorespiratory reflexes in diabetes mellitus. Diabetes Care.

[B29] Waterman SA (2001). Autonomic dysfunction in Lambert-Eaton myasthenic syndrome. Clin Aut Res.

[B30] Bassotti G, Villanacci V, Mazzocchi A, Castellani D, Giuliano V, Corsi S, Morelli A (2006). Colonic propulsive and postprandial motor activity in patients with ulcerative colitis in remission. Eur J Gastroenterol Hepatol.

